# Phase-Lock Requirements in a Serial Array of Spin Transfer Nano-Oscillators

**DOI:** 10.1038/srep11462

**Published:** 2015-06-18

**Authors:** T. Qu, R.H. Victora

**Affiliations:** 1 School of Physics and Astronomy, University of Minnesota, Minneapolis, Minnesota 55455, USA; 2 Department of Electrical and Computer Engineering, University of Minnesota, Minneapolis, Minnesota 55455, USA

## Abstract

The most promising approach to attain a narrow linewidth and a large output power simultaneously in spin torque oscillators is self-phase-locking of an array of oscillators. Two long range coupling mechanisms, magnetostatic interaction and self-induced current, are explored. Synchronization occurs with MR ratio ~14% and volume ~2.1 × 10^−5^ *μm*^3^ at room temperature for an experimental frequency dispersion, when only the self-induced microwave current is present. The dipole interaction decreases the MR ratio requirement when the elements are properly spaced.

The prediction of magnetization manipulated through a spin polarized electric current^1^,^2^ has stimulated tremendous research interest in recent years^3^. The current-induced stable magnetization precession device, named the spin torque nano-oscillator (STNO), provides the first alternative to a standard LC-tank voltage-controlled oscillator (VCO) and shows intriguing advantages of versatile frequency tunability, nanoscale size, broad working temperature, and easy integration with standard silicon technology over a VCO. These characteristics make STNO a very attractive novel device for future applications, e.g. chip-to-chip micro-wireless communications.

The system including a single STNO has been studied extensively experimentally^4^,^5^ and theoretically^6^. However, the power from a single STNO remains in the low nW range^4^, but applications would greatly benefit from microwatt power levels. Although a magnetic tunnel junction (MTJ) may produce a large voltage signal benefiting from large magnetoresistance^7^ to 400% at room temperature, the maximum bias current is limited by the barrier breakdown voltage (~1.0 V) and thus the power is limited to sub microwatt^8^. Also, the linewidth as a measure of the phase noise in MTJ is rather large(~100 MHz). One approach to achieving a narrow linewidth and a large output power simultaneously is the excitation of vortex dynamics in a MTJ or metallic spin-valve but the oscillation frequency is limited to sub-GHz range^9^. Another approach is to synchronize arrays of STNOs by local or non local mechanisms. The spin-wave coupling in point-contact geometry only weakly couples more than two closely spaced STNOs because of the fractional oscillator distance relative to the spin wave length^10^,^11^. Besides, the coupling length is only efficient over the spin wave decay length, i.e., around 1 *μ*m. Thus, the most promising approach is a long range coupling through STNOs’ self-emitted microwave currents, predicted by Grollier, Cros and Fert^12^. They argued that the oscillators of an electrically connected network could be mutually phase locked through the microwave feedback current, although they neglected thermal noise and long range magnetostatic interaction. As the coupling strength is proportional to the microwave current, it is imperative to gain insight into the magnetoresistance(MR) ratio necessary for synchronization. This will allow the design and optimization of the STNO-based spintronic device. The previously neglected thermal noise produces a broadened peak and is detrimental to reaching a phase-lock state, while in contrast, the magnetostatic field could enhance the coupling.

In this paper, we use the Landau-Lifschitz-Gilbert equation to describe the dynamics of each individual oscillator and study in detail the MR ratio threshold necessary to achieve phase-locking under two common magnetic nonuniformities: variation in the anisotropy and saturated magnetization magnitude. We check the effect of thermal fluctuation on the required minimum MR ratio. The interaction between the microwave current coupling and the long range coupling mechanism provided by the magnetostatic field is also studied. The interaction effect on the MR ratio threshold is obtained.

## Results & Discussion

**Figure 1:**
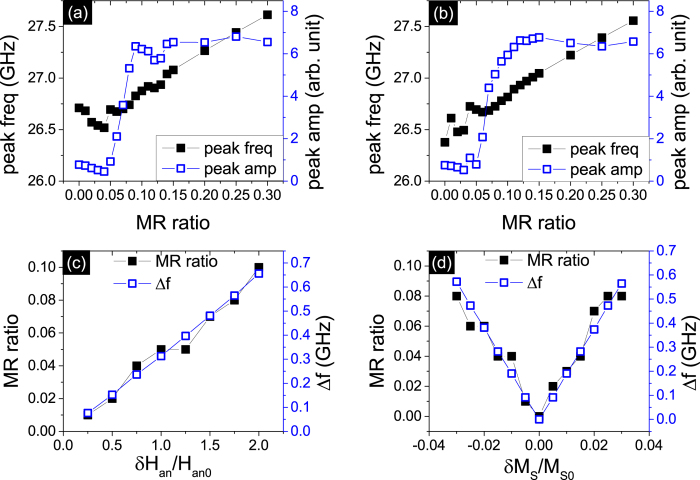
(**a,b**) The peak frequency and peak amplitude of the voltage signal versus MR ratio for the 10 non-uniform serial STNOs under the conditions of (**a**) *δ*H_*an*_/H_*an*0_ = 1.8 and (**b**) *δ*M_*S*_/M_*S*0_ = 0.03 without thermal fluctuation. Both the MR ratio thresholds are 8%. The peak frequency and amplitude at MR ratio = 0% are the mean values from 10 separate peaks. (**c,d**) The MR ratio threshold and frequency dispersion versus (**c**) *δ*H_*an*_/H_*an*0_ and (d) *δ*M_*S*_/M_*S*0_.

Figure 1(a,b) show the sharp transition from distinct oscillation states to synchronized states, occurring for dispersed anisotropies or dispersed saturation magnetizations in stacked STNOs. The anisotropy and magnetization dispersion follows the same rule: H_*an*_ = H_*an*0_ + (i − 1)/(N − 1) × *δ*H_*an*_ (or M_*S*_ = M_*S*0_ + (i − 1)/(N − 1) × *δ*M_*S*_), with i varying between 1 and 10. *δ*H_*an*_(*δ*M_*S*_) is defined as the amount of anisotropy(saturation magnetization) difference between consecutive free layers. Under both conditions, the synchronization process begins when the MR ratio is above 5%. The MR ratio threshold MR_*th*_ to achieve the completely synchronized state is 8%, while below this threshold, the total oscillation state is partially synchronized with individual adjacent peaks merged into several multiple peaks. The frequency increases monotonically in the MR ratio range where the synchronization mechanism occurs. This trend may be caused by the positive dc component in the self-generated microwave current. In the synchronized state, the peak is similar to a delta function in that its linewidth is nearly zero. The amplitude has a small oscillation with MR ratio caused by the finite integration time (8.4 ms). The time scale of the dynamics for the transition from the static state to synchronization is about one nanosecond for the different MR ratios above the threshold. We also check the MR_*th*_ as a function of *δ*H_*an*_ and *δ*M_*S*_ as shown in Fig. 1(c,d). The threshold is linearly dependent on the *δ*H_*an*_ and *δ*M_*S*_. The frequency range Δf, defined as the intrinsic frequency difference of N oscillators, is induced by the non-uniform properties of H_*an*_ or M_*S*_: it is found that the MR_*th*_ is also linearly dependent on the Δf. Although the coupling appears to be more difficult under the condition of saturated magnetization variation, considering its influence on both the excited oscillation energy and the microwave feedback, the MR_*th*_ can be viewed as determined only by the resulting frequency range. Based on this prediction, the observed experimental frequency dispersion^10^ of 1.25% needs about 4.7% MR ratio to achieve phase-lock, which is achievable in a CPP-GMR structure.

**Figure 2:**
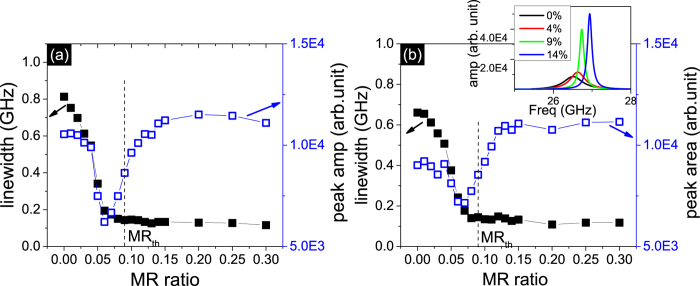
(**a,b**) The linewidth and peak area of the maximum peak in the voltage signal spectrum versus MR ratio for the 10 non-uniform serial STNOs under the condition of (**a**) *δ*H_*an*_/H_*an*0_ = 1.8 and (b) *δ*M_*S*_/M_*S*0_ = 0.03 at T = 50 K. The corresponding MR ratio thresholds are both 9%. The inset of (**b**) shows the spectra of the amplitude versus frequency for the set of 10 oscillators when the MR ratio is 0, 4%, 9% and 14% for the first order excitation frequency.

**Figure 3:**
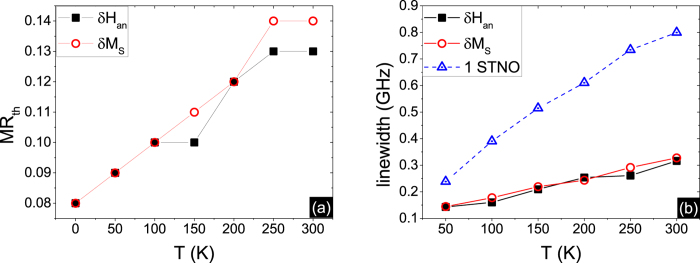
(**a**) MR ratio threshold for the array of 10 non-uniform serial STNOs under the conditions of *δ*H_*an*_/H_*an*0_ = 1.8 and *δ*M_*S*_/M_*S*0_ = 0.03 at variable temperatures. (**b**) The linewidth of the voltage signal generated by 10 oscillators at the MR_*th*_ at variable temperature under the corresponding anisotropy and magnetization saturation conditions. The linewidth of a single oscillator is shown for comparison.

While the noise in the magnetic system caused by thermal fluctuations is detrimental to phase locking, the self-generated ac current reduces the incoherent phase found in multiple oscillator states. This helps to enhance the peak height and reduce the peak linewidth, as shown in the inset of Fig. 2(b). The linewidth decreases to a minimum value when the array of oscillators achieves a completely synchronized state, in Fig. 2. The threshold MR obtained increases with temperature under both conditions of M_*S*_ and anisotropy dispersion, as shown in Fig. 3(a). The dependence on temperature is approximately linear for the volume considered (~2.1 × 10^−5^ *μm*^3^). The MR_*th*_ for *δ*H_*an*_/H_*an*0_ = 1.8 and *δ*M_*S*_/M_*S*0_ = 0.03 is 13% and 14% respectively at room temperature 300 K. We inquire into the peak profile at these threshold points and compare the phase-locked state of N oscillators with one single oscillator, in Fig. 3(b). This self-generated microwave is quite efficient in decreasing the noise at room temperature, and the peak of the array of oscillators is tremendously narrowed compared with the single STNO.

**Figure 4:**
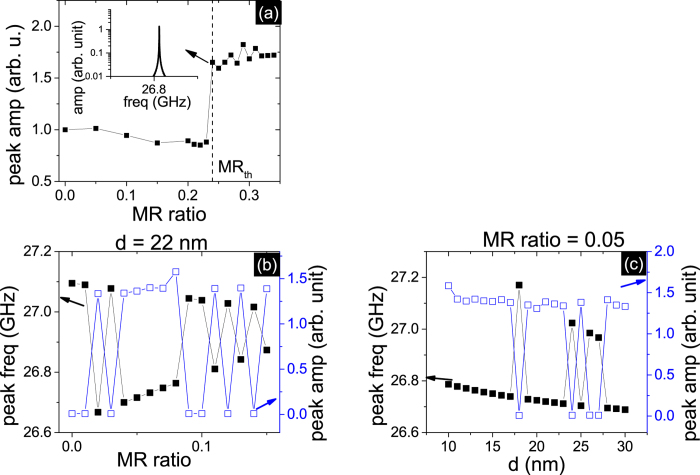
(**a**) The peak amplitude of the voltage signal versus MR ratio for 2 non-uniform serial STNOs under the condition of *δ*H_*an*_/H_*an*0_ = 1.8 at T = 0 K, when only the current feedback mechanism is included. The inset is the spectrum of the amplitude vs frequency at the MR ratio threshold of 24%. (**b**)The peak frequency and amplitude versus MR ratio when the distance d between two free layers is fixed at 22 nm. (**c**) The peak frequency and amplitude versus distance d when MR ratio of the STNOs is fixed at 0.05. In both (**b**,**c**), the combined mechanisms of current and magnetic feedback are considered.

Magnetic feedback from the time dependent dipole field between the STNOs could enhance the coupling at a proper distance and reduce the MR ratio requirement for the phase locked state. We use the finite difference integration technique (more than 7000 elements per oscillators) to calculate the magnetostatic interaction. We study the coupling between two non-identical oscillators with *δ*H_*an*_/H_*an*0_ = 1.8 at T = 0 K. When only microwave current is included, the MR ratio threshold is 24% in Fig. 4(a). This is higher than the case of 10 oscillators because 2 oscillators generate less ac current. When the ac dipole field between two oscillators is included at a distance d of 22 nm (d is the distance between two oscillation layers in the two STNOs), the phase locked state could be achieved at a low MR ratio of 2%. Under this MR ratio, the ac current is not sufficient to reach synchronization using only the current coupling mechanism. Thus the assistance from the ac dipole field is important to the in-phase synchronization. However, the combined mechanisms of magnetic and electric feedback is more complicated than the electric feedback mechanism alone. We observe oscillatory behavior in Fig. 4(b) of the predicted peak amplitude versus MR ratio at a fixed d of 22 nm under the combined mechanisms, while for the electric feedback alone, the two oscillations stay coupled in phase after overcoming the MR ratio threshold. This oscillatory behavior also happens when varying d at a constant MR ratio of 0.05, shown in Fig. 4(c).

**Figure 5:**
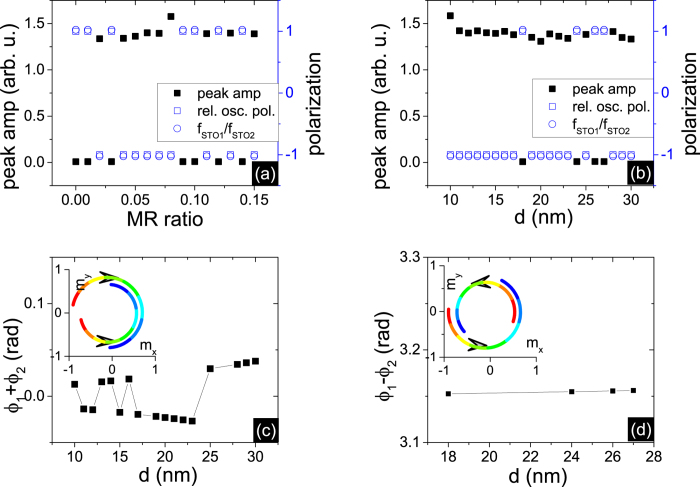
The peak amplitude, relative oscillation polarization of the output voltage signal and the ratio *f*_*STO*1_/*f*_*STO*2_ of estimated intrinsic frequencies of 2 non-uniform STNOs (**a**)versus MR ratio at d = 22 nm (**b**) versus distance d at MR ratio = 0.05 under the condition of *δ*H_*an*_/H_*an*0_ = 1.8 at T = 0 K. The relative oscillation polarization is calculated as polarization of *STO*_1_ over polarization of *STO*_2_. (**c**) the phase sum *φ*_1_ + *φ*_2_ versus d when the polarization state in fig(b) is opposite. (**d**) the phase difference *φ*_1_ − *φ*_2_ versus d when the polarization state in fig(b) is the same. The insets shows the time evolution projection into the xy plane of the normalized magnetization vector of 2 STNOs for nearly one period. The color depicts the time: red refers to the initial time while blue refers to the final time. The projection of *STO*_2_ is reduced to reveal the phase difference of the two oscillators.

To analyze the complex oscillatory behavior, we investigate the out-of-plane oscillation modes 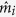 = (r_*i*_cos(*φ*_*i*_), r_*i*_sin(*φ*_*i*_), m_*iz*_), where r_*i*_ and *φ*_*i*_ is the amplitude equal to 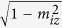 and the phase of the i (i = 1,2) oscillator and m_*iz*_ is a constant. By Fourier transform of 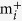 = m_*ix*_ + *im*_*iy*_, we obtain the polarization of the out-of-plane oscillation mode. The polarization is defined as +1 for counterclockwise rotation, and the polarization is −1 for the converse case. We find that for small output power, the two oscillators are in the same polarizations, while for large power, the two oscillators have opposite polarizations, shown in Fig. 5(a,b). Small power, which decreases nearly to 0, implies the m_*ix*_ oscillation is out of phase, as shown in the insets. We examine the phase difference *φ*_1_ − *φ*_2_ and it closely equals to *π* when the two oscillators have the same polarization in Fig. 5(d). For the opposite polarization states, the phase sum *φ*_1_ + *φ*_2_ is approximately 0 in Fig. 5(c), which implies that the m_*ix*_ component is in phase and the m_*iy*_ component is out of phase.

To illustrate the synchronization via the ac dipole field and self-generated microwave current, we study the phase dynamics of the STNOs in the extended Kuramoto model^6^,^13^. The energy E_*i*_ injected into the individual oscillator from the feedback mechanisms is computed in one period. Here, the spin torque field produced by the ac current is neglected, because its amplitude is less than 0.01 of the dipole field 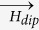 amplitude.


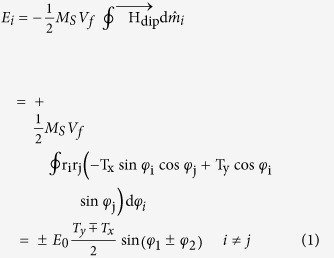


Here, 
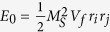
. *T*_*x*_ and *T*_*y*_ are the diagonal elements of the magnetostatic tensor. The sign of ± corresponds to the opposite (same) polarization. Following references 6 and 16, energy *E*_*i*_ could be treated as a perturbation, compared to the excited state energy *E*_0*i*_ ~ 2*π*(*M*_*S*_*m*_*iz*_)^2^. The modified frequency *δf*_*i*_ due to 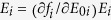. Thus the phase dynamics for the STNOs are


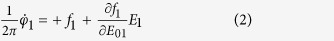



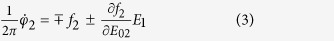


**Figure 6:**
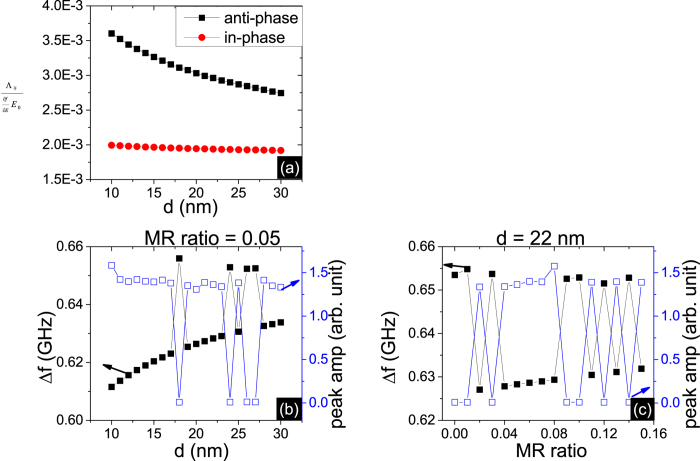
(**a**) Calculations of coupling parameters 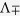 using Eq.(4) for the two polarization states, taking the phase data from the simulation result. Estimated intrinsic frequency difference Δ*f* and the peak amplitude of the output voltage signal (**b**) versus d at MR ratio = 0.05 (**c**) versus MR ratio at d = 22 nm.

For the synchronized STNO array, both oscillators are in resonance frequency *f*_*res*_. Thus the coupling parameter, which controls the frequency range consistent with synchronization, is


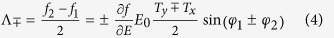



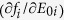
 is treated as the same constant for the two STNOs in the first order estimation. From the 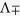 in Fig 6(a), we deduce a conclusion that the case of opposite polarization has weaker coupling than the case of same polarization because of the opposing effects of *T*_*y*_ and *T*_*x*_. So if the intrinsic frequency difference Δ*f* = *f*_2_ − *f*_1_ exceeds the coupling strength for opposite polarization, the same polarization is chosen to achieve the resonant but anti-phase state. 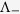 also predicts the opposite polarization state is impossible for a STNO array of circular shape. It has no capability of coupling the non-uniform oscillators as *T*_*x*_ completely cancels *T*_*y*_. We find that in the resonant state of two STNOs of circular shape, anti-phase out-of-plane oscillation is always preferred.

To confirm the coupling parameters analysis, we estimate the intrinsic frequency difference Δ*f*. The approximate intrinsic frequency *f*_*i*_ equals to 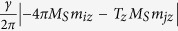. Here, *T*_*z*_ is the diagnonal element of the magnetostatic tensor and negative. If the z components of two STNOs have opposite signs, which represent the out-of-plane direction of the oscillation modes, Δ*f* equals to 
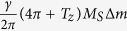
. For the same sign of *m*_*iz*_, Δ*f* equals to 
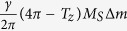
. It is reasonable to treat Δ*m* unchanged in both polarization states in the perturbation scheme. Thus the oscillation state with the same sign of *m*_*iz*_ has larger intrinsic frequency difference. From the simulation results, Δ*f* under the same sign of *m*_*iz*_ is in the range of 0.65 to 0.67 GHz and needs more coupling to lock phase, compared with Δ*f* under the case of the opposite signs in the range of 0.61 to 0.63, in Fig. 6(b,c). Thus the relative direction of *m*_*iz*_ chooses the polarization state directly to be the same or opposite. This connection is verified for both conditions of fixed distance or fixed MR ratio, as shown in Fig. 5(a,b). The *m*_*iz*_ evolution is determined interactively by magnetostatic and current feedback mechanism and is difficult to predict. Therefore device design may require a technique for selecting the starting point in order to guarantee the same phase locked state for each use.

## Conclusion

In conclusion, we obtain the minimum MR ratio requirement for synchronization subject to the two long range coupling mechanisms of self-induced microwave current and dipole field. The MR ratio threshold is determined by the frequency dispersion (caused by non-uniform properties). It increases linearly as thermal fluctuations induce chaos in the serial array of STNOs. At room temperature, the MR ratio requirement is achievable in GMR devices under the experimental frequency dispersion. When the interaction between self-induced dipole field and microwave current is included, the set of oscillators shows oscillatory phase behavior between *π* and 0 phase when the dipole field or MR ratio is varied. The proper coupling interaction benefits the synchronization so that the required MR ratio for synchronization is below the value for coupling by current alone.

## Method

Magnetization dynamics in the nanoscale can be accurately described using the Landau-Lifschitz-Gilbert equation.


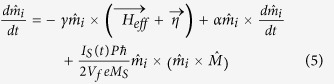


where *γ*, *α*, and 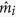 are the gyromagnetic ratio, the damping constant, and the unit vector of the free layer magnetization of the ith STO (i = 1:10). The effective field 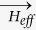 includes the external field, the magnetocrystalline anisotropy and any magnetostatic contributions that may be present. Both the external and anisotropy field are in the x direction. 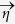 is the thermal fluctuation field^14^, described by a white noise with amplitude dependent on the temperature and the free layer volume V_*f*_. The third term on the right is the spin torque generated by the spin current. The current is polarized by the fixed layer magnetization 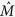, in the +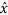 direction. *ħ* is Plancks constant, e is the magnitude of the electron charge, P is the polarization constant and M_*S*_ is the free layer saturation magnetization. I_*S*_(*t*) includes the dc source current and ac self-generated current. Under the first order estimation of the circuit^12^,


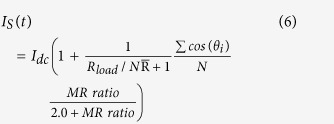


N is the number of oscillators. *θ*_*i*_ is the angle between the magnetization in the free layer and fixed layer. R_*load*_ is 50 Ω for the bias tee in the circuit^15^. R = (R_*P*_ + R_*AP*_)/2 is the average resistance of the parallel and anti-parallel states of a single STNO where R_*P*_ = 10 Ω. The material parameters for Co in the following calculations are *α* = 0.007, P = 0.35, M_*S*_ = 1352 emu/cm^3^, H_*an*_ = 500 Oe. H_*ext*_ = 2000 Oe and the current is 9 mA, that the current density is in the order of 10^7^ A/cm^2^ to 10^8^ A/cm^2^.

**Figure 7:**
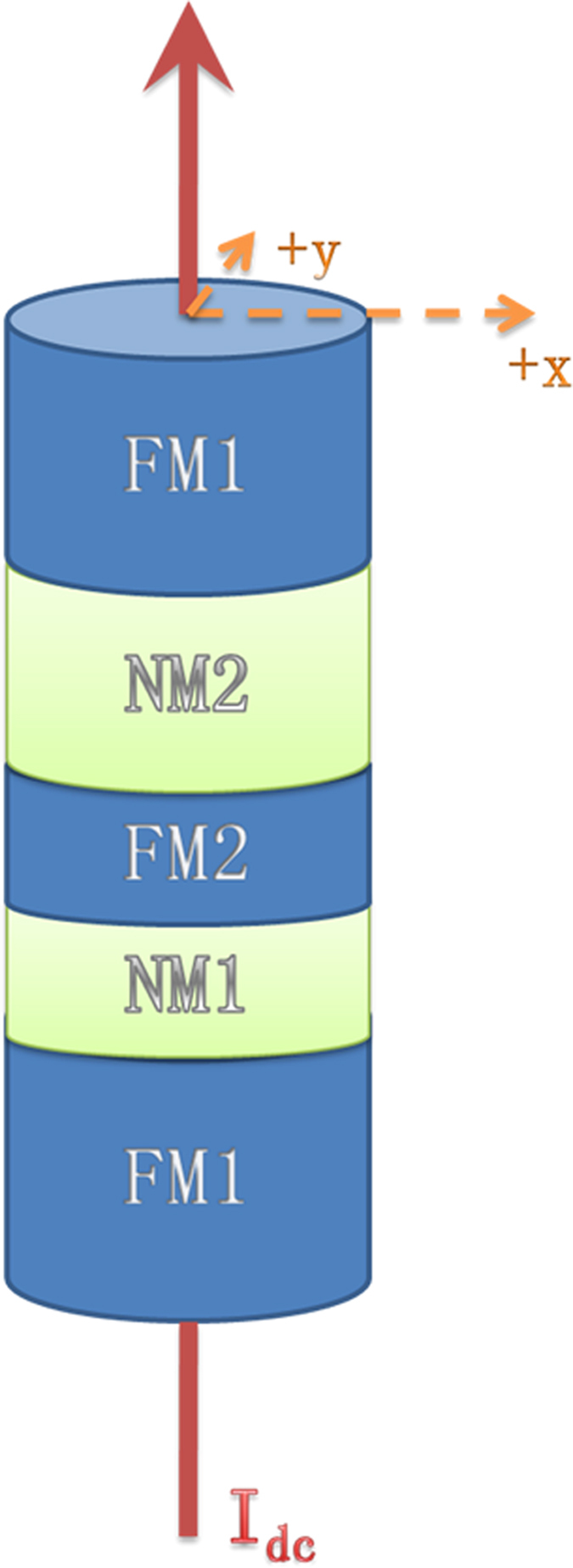
The geometry of STOs in an elliptically shaped nanowire. This structure is repeated to implement a serial array of STOs. The current is perpendicular to the plane. The x axis is along the major axis and y axis is along the minor axis of the ellipse.

The geometry of serially connected STOs could be implemented in the type of nanowires which have been developed for CPP-GMR experiments^16^. The standard structure is a multilayer nanowire composed of ferromagnetic(FM) and normal metal(NM) layers, such as [FM1(fixed)/NM1/FM2(free)/NM2]_*N*_, where N is the number of oscillators. When the magnetization in FM2 layer is excited to a stable oscillation state, FM1 functions as both a polarizer of injected charge current depolarized by NM2 and as an analyzer to record the generated voltage signal. Positive injected dc current is defined as electrons flowing from the free to the fixed magnetic layer. The free layer is assumed to be a typical Co thin film with a thickness of 3 nm and an elliptical shape of dimensions 130 nm × 70 nm, shown in Fig. 7. In this case, at least 10 coupled oscillators are required to produce power approaching *μ*W, for a reasonable MR range of 1 ~ 10%.

## Additional Information

**How to cite this article**: Qu, T. and Victora, R.H. Phase-Lock Requirements in a Serial Array of Spin Transfer Nano-Oscillators. *Sci. Rep.* **5**, 11462; doi: 10.1038/srep11462 (2015).
